# Comparative Study of the Immune Microenvironment in Heterotopic Tumor Models

**DOI:** 10.3390/cancers16020295

**Published:** 2024-01-10

**Authors:** Melanie Kienzl, Kathrin Maitz, Arailym Sarsembayeva, Paulina Valadez-Cosmes, Eva Gruden, Dusica Ristic, Karolina Herceg, Julia Kargl, Rudolf Schicho

**Affiliations:** 1 Division of Pharmacology, Otto Loewi Research Center, Medical University of Graz, 8010 Graz, Austria; 2 BioTechMed, 8010 Graz, Austria

**Keywords:** tumor microenvironment, immune composition, KP, LLC, MC38, B16-F10

## Abstract

**Simple Summary:**

The body’s defense system, which comprises immune cells, is an important factor contributing to tumor growth and treatment response. A “hot” tumor, infiltrated by various beneficial immune cells, usually corresponds with a better prognosis. A so-called “cold” tumor that lacks these beneficial immune cells or that contains harmful cells that block an immune response often results in a harder fight against cancer. Turning a “cold” tumor into a “hot” tumor may help cancer treatments, like immunotherapy, work better. In this study, we looked at the immune cell environment in lung, colorectal, and skin cancer. Tumors were grown in mice, and the different types of immune cells were studied using a fluorescent technique. We found differences in the immune cell infiltration of the diverse tumor types, with some tumors being more “hot” and others showing a tendency to be “cold”. These findings can help us understand how various tumor types interact with the immune system.

**Abstract:**

The tumor microenvironment (TME) is pivotal in cancer progression and the response to immunotherapy. A “hot” tumor typically contains immune cells that promote anti-tumor immunity, predicting positive prognosis. “Cold” tumors lack immune cells, suggesting a poor outlook across various cancers. Recent research has focused on converting “cold” tumors into “hot” tumors to enhance the success of immunotherapy. A prerequisite for the studies of the TME is an accurate knowledge of the cell populations of the TME. This study aimed to describe the immune TME of lung and colorectal cancer and melanoma, focusing on lymphoid and myeloid cell populations. We induced heterotopic immunocompetent tumors in C57BL/6 mice, using KP and LLC (Lewis lung carcinoma) cells for lung cancer, MC38 cells for colorectal cancer, and B16-F10 cells for melanoma. Immune cell infiltration was analyzed using multicolor flow cytometry in single-cell suspensions after tumor excision. KP cell tumors showed an abundance of neutrophils and eosinophils; however, they contained much less adaptive immune cells, while LLC cell tumors predominated in monocytes, neutrophils, and monocyte-derived dendritic cells. Monocytes and neutrophils, along with a significant T cell infiltration, were prevalent in MC38 tumors. Lastly, B16-F10 tumors were enriched in macrophages, while showing only moderate T cell presence. In conclusion, our data provide a detailed overview of the immune TME of various heterotopic tumors, highlighting the variabilities in the immune cell profiles of different tumor entities. Our data may be a helpful basis when investigating new immunotherapies, and thus, this report serves as a helpful tool for preclinical immunotherapy research design.

## 1. Introduction

Rudolf Virchow was the first to identify leukocytes in cancerous tissue in the late 19th century [1]. However, it was not until the early 21st century that the interplay of tumor cells with leukocytes was finally acknowledged as an important factor in tumor progression [2]. Gene mutations (due to (epi)-genetic and environmental factors) are major drivers of tumorigenesis [3,4], but aberrant cell growth can be recognized and controlled by the immune system through a process called immune surveillance [5]. However, malignant cells acquire traits, such as the loss of immunogenic features or the mechanisms of immune suppression, leading to immune escape and tumor progression [6].

Nonetheless, next to tumor cells, immune cells as well as non-immune stromal cells represent important components of a niche in tumors, called the tumor microenvironment (TME), which has emerged as an essential player of tumor progression [7]. The TME is classified as a tumor hallmark with complex cellular composition [2]. It continuously evolves during tumorigenesis and differs significantly between tumor entities, significantly contributing to patient outcomes [8,9]. Especially, the composition of the infiltrated immune cells is crucial for tumor development [10]. As such, the so-called “hot” tumors are infiltrated by anti-tumorigenic leukocytes, including CD8^+^ T cells and natural killer (NK) cells, whereas the so-called “cold” tumors are either immune excluded (lacking the infiltration of leukocytes) or infiltrated by more pro-tumorigenic leukocytes, including neutrophils, monocytes, and regulatory T cells (T regs) [11]. However, it must be emphasized that the characterization of these cells as pro- or anti-tumorigenic is not black and white. It may depend, amongst other factors, on the tumor entity, soluble mediators, cell–cell interactions, the immune evading/suppression mechanisms of a tumor [8,10,12], and how immune cells behave in the TME.

In the present study, we aimed at characterizing and comparing the immune cell profiles in tumors of lung and colon cancer as well as melanoma models. According to the estimated 2023 cancer statistics by Siegel and colleagues, lung cancer is the second most common type of cancer in terms of new cases, following prostate/breast cancer, with colorectal cancer at the third and melanoma at the fifth place in the US [13]. Additionally, lung cancer is recognized as the cancer with the most estimated deaths, closely followed by colorectal cancer [13], despite the use and development of new therapies.

In recent years, understanding the TME has gained importance for the development of new cancer therapies, such as immune checkpoint inhibitor therapies [14], which were shown to be successful in solid tumor entities [15]. They have been included into the standards of care of non-small cell lung cancer, colorectal cancer, and melanoma [16,17,18]. However, the rate of non-responders remains high [19,20]. Importantly, the composition of immune cell in the TME has profound influence on the therapy response in certain patients [21]. As such, it has been described that a “hot” tumor shows a better response towards immune checkpoint inhibitor therapy when compared to a “cold” tumor [22]. Thus, an improved understanding of how the immune system interplays with tumor cells in the TME is essential for developing new and effective (adjuvant) immunotherapies.

Therefore, we performed a comprehensive comparison of the immune TME of tumors derived from lung, colon, and skin cancer cell lines using immunocompetent heterotopic mouse models and multicolor flow cytometry. We aimed at highlighting the composition of immune cells and their subsets in these cancer entities to provide a basis for further research on immune cell involvement in tumor progression. We identified substantial variances in the viability of the TME cells as well as in the immune profiles of the investigated TMEs.

## 2. Materials and Methods

### 2.1. Cell Lines and Cell Culture

The murine KP cell line was isolated from a lung adenocarcinoma of a Kras^LSL−G12D^/p53^fl/fl^ mouse at the Fred Hutchinson Cancer Center (Seattle, WA, USA) after the intratracheal administration of adenoviral Cre recombinase as described before [23]. The cell line was generously provided by Dr. McGarry Houghton. LLC, B16-F10, and MC38 cells were purchased from ATCC. The KP, LLC, B16-F10, and MC38 cells were maintained in DMEM with 10% FBS (Life Technologies, Vienna, Austira) and 1% penicillin/streptomycin (P/S, PAN-Biotech, Aidenbach, Germany) at 37 °C and 5% CO_2_ in a humidified atmosphere. 

### 2.2. Animal Studies and Tumor Models

All animal experiments were performed in the animal facilities of the Medical University of Graz. C57BL/6 mice were purchased from Charles River and bred in house. Approval for animal experimental protocols was granted by the Austrian Federal Ministry of Science and Research (protocol number: BMBWF-66.010/0041-V/3b/2018).

LLC, B16-F10, MC38, KP (all 0.5 × 10^6^), and CT26 cells (0.1 × 10^6^) were subcutaneously (s.c.) injected into the right flank of mice. Tumor growth was monitored during the course of the experiments. Mice were sacrificed after two to three weeks, and tumors were subsequently collected [24].

### 2.3. Preparation of Single-Cell Suspensions

The preparation of single-cell suspensions from tumors was performed as previously described [24]. Using a scalpel, tumors were minced and digested with DNase I (160 U/mL; Worthington Biochemical Corporation, Lakewood, NJ, USA) and collagenase (4.5 U/mL; Worthington Biochemical Corporation) for 25 min at 37 °C while rotating at 1000 rpm. After incubation, they were passed through a 40 µm cell strainer, washed in PBS+2% FBS, counted, resuspended in PBS, and used for antigen staining. The s.c. tumors of B16-F10 cells were processed without enzymatic digestion and only directly passed through a 40 µm cell strainer, before washing, counting, and antigen staining.

### 2.4. Flow Cytometric Phenotyping of Immune Cell Populations

First, single-cell suspensions were incubated for 20 min in Fixable Viability Dye (FVD) eFluor^TM^ 780 (eBioscience, Thermo Fisher Scientific, Waltham, MA, USA) in the dark to exclude dead cells. After adding 1 µg of TruStain^TM^ FcX (Biolegend, San Diego, CA, USA), immunostaining was performed on ice for 30 min (protected from light) with the following antibodies: CD45-AF700 (# 103128), CD45-BV785 (# 103149), Ly6C-APC (# 128015), Ly6G-PE/Dazzle594 (# 127648), CD11c-BV605 (# 117334), CD8-PerCPCy5.5 (# 100734), CD62L-BV605 (# 104438), NKp46-BV510 (# 137623), CD19-FITC (# 115506), CD62L-BV605 (# 104438), CD103-BV510 (# 121423), MHC-II-PerCPCy5.5 (# 107625), and CD206-FITC (# 141703) (all antibodies from Biolegend); CD11b-BUV737 (# 612801), F4/80-BUV395 (# 565614), Siglec-F-PE (# 552126), CD3-BUV395 (# 563565), CD4-BUV496 (# 564667), CD44-BUV737 (# 612799), and gdTCR-PECF594 (# 563532) (all antibodies from BD Biosciences, Franklin Lakes, NJ, USA); and FoxP3-PE (# 12-5773-82) from eBioscience. After staining, cells were washed and fixed using IC Fixation Buffer (eBioscience). For nuclear antigen staining, cells were permeabilized with Transcription Factor Buffer Set (BD Biosciences, # 562574) prior to staining with nuclear antibodies. Samples were stored at 4 °C in staining buffer until they were analyzed on a BD LSRFortessa^TM^ flow cytometer with FACSDiva software (v9.0.1, BD Biosciences). Analyses and compensation were performed with Flowjo software (Version 10.8.1, TreeStar, BD Biosciences). Fluorescence minus-one-samples were used to define gates. 

### 2.5. Statistical Analysis

Statistical analyses for in vivo experiments were performed using GraphPad Prism 10.0.3 (GraphPad Software, Boston, MA, USA). Significant differences between four experimental groups were determined using one-way ANOVA with Tukey’s post hoc test for corrections of multiple comparisons. A *p*-value of <0.05 indicated statistical significance.

## 3. Results

### 3.1. Subcutaneous Tumors of Lung Cancer, Colon Cancer, and Melanoma Differ in Their Viability and Immune Cell Composition

To characterize the TME in different tumor entities, we subcutaneously (s.c.) injected C57BL/6 mice with lung cancer (LLC, KP), colon cancer (MC38), and melanoma (B16-F10) cell lines. After tumors grew to a certain volume, mice were sacrificed, and tumors were excised. Subsequently, the single-cell suspensions of the tumors were prepared, stained with antibodies, and analyzed using multicolor flow cytometry.

**Figure 1:**
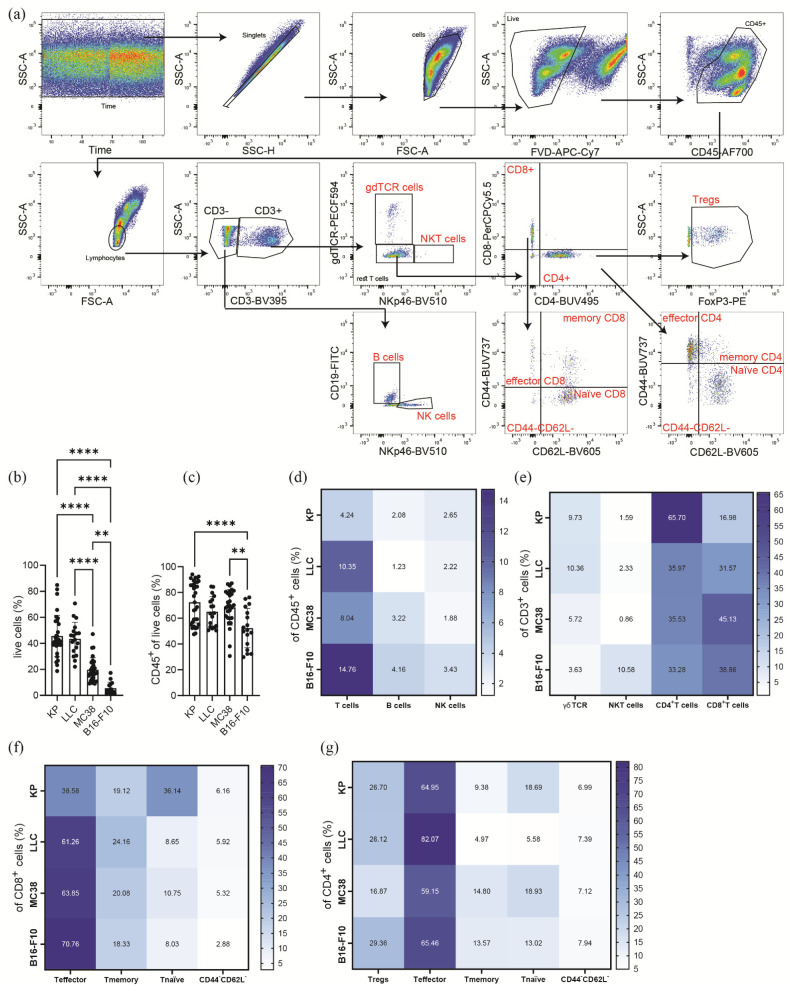
Lymphoid immune microenvironment of heterotopic tumor models. (**a**) Representative gating strategy, of KP tumor single-cell suspension, for the analysis of the lymphoid immune microenvironment of single-cell suspensions in heterotopic tumors. Tumor infiltrating leukocytes (CD45^+^) were pre-gated for time and singlets. Dead cells were excluded by using a fixable viability dye (FVD). A lymphocyte size gate was used to pre-gate the lymphoid populations. T cells were gated as CD45^+^/CD3^+^; NK cells as CD45^+^/CD3^−^/NKp46^+^; B cells as CD45^+^/CD3^−^/CD19^+^; CD8^+^ T cells as CD45^+^/CD3^+^/CD8^+^; CD4^+^ T cells as CD45^+^/CD3^+^/CD4^+^; and regulatory T cells (T regs) as CD45^+^/CD3^+^/CD4^+^/FoxP3^+^. NKT cells were gated as CD45^+^/CD3^+^/NKp46^+^ and γδT cells as CD45^+^/CD3^+^/gdTCR^+^. To characterize effector (T effector, CD44^+^CD62L^−^), memory (T memory, CD44^+^/CD62L^+^), naϊve (T naïve, CD44^−^/CD62L^+^), and CD44^−^CD62L^−^ subsets in T cells, CD4^+^ and CD8^+^ T cells were further gated. (**b**–**g**) Flow cytometric analysis of single-cell suspensions of s.c. tumors of lung cancer cell lines (KP, *n* = 32; LLC, *n* = 18), colon cancer (MC38, *n* = 28), and melanoma (B16-F10, *n* = 17). Data were pooled from 2 to 5 independent experiments. The percentage of live (**b**) and CD45^+^ (**c**) cells is shown. (**d**–**g**) Heatmaps show percentage of CD45^+^ (**d**), CD3^+^ (**e**), CD8^+^ (**f**), and CD4^+^ (**g**) cells measured in tumors, ranging from the highest (dark blue) to the lowest numbers (white), respectively. Statistical differences were assessed by using one-way ANOVA with Tukey’s post hoc test. ** *p* < 0.01; **** *p* < 0.0001.

First, we focused on the general viability and the lymphoid subsets of the infiltrating cells. The gating strategy for the lymphoid panel can be found in Figure 1a. The viability of single-cell suspensions was similar between tumors derived from the lung cancer cell lines (KP and LLC, >40%); however, it was significantly reduced in MC38 cell tumors (20%). In contrast, only about 5% of cells in the single-cell suspensions of B16-F10 tumors were viable (Figure 1b). The general infiltration of leukocytes into tumors was measured by analyzing CD45^+^ cells. The frequencies of CD45^+^ cells were >64% for KP, LLC, and MC38 cell line tumors, whereas in B16-F10 tumors, the frequency was slightly lower, only showing 54% CD45^+^ cells (Figure 1c).

### 3.2. Subcutaneous Tumors of Lung Cancer, Colon Cancer, and Melanoma Show Significantly Different Lymphoid Immune Cell Compositions of the TME

When looking at the major lymphoid cell subsets, we identified a significantly elevated infiltration of CD3^+^ T cells in the TME of B16-F10 tumors. T cell infiltration was reduced in LLC and MC38 tumors, and even further reduced in KP tumors (Figure 1d). With regard to B cell and NK cell infiltration, there were no significant differences between the investigated tumor entities (Figure 1d, Supplementary Figure S1a, Supplementary Table S1).

In Figure 1e (Supplementary Figure S1b, Supplementary Table S2), we identified differences in the CD3^+^ subpopulations between all investigated tumor entities. γδTCR^+^ cells were significantly reduced in the TME of MC38 and B16-F10 tumors as compared to the lung cancer TME. In contrast to the lung and colon cancer TME, there was an increased infiltration of NKT cells (NKp46^+^ CD3^+^ cells) into B16-F10 tumors. The frequency of CD4^+^ T cells was approximately double in KP tumors (<60%) when compared to the other cell lines (>30%). In contrast, CD8^+^ T cells were significantly reduced in the KP TME (<20%) when compared to the TMEs of LLC, MC38 and B16-F10 cell tumors.

Regarding the activation status of CD8^+^ T cells, we noticed that KP cell tumors contained significantly decreased frequencies of effector CD8^+^ T cells (characterized by CD44^+^CD62L^−^). However, compared to LLC, MC38, and B16-F10 cell tumors (Figure 1f, Supplementary Figure S1c, Supplementary Table S3), KP cell tumors showed significantly increased numbers of naϊve CD8^+^ T cells (CD44^−^CD62L^+^). No differences between the TMEs were observed for memory CD8^+^ T cells (CD44^+^CD62L^+^); a significant decrease in CD44^−^CD62L^−^ CD8^+^ T cells was observed in B16-F10 cell tumors as compared to KP cell tumors. Regarding the frequencies of T regs, they were significantly reduced in MC38 cell tumors by approximately one-third when compared to the other tumor entities (Figure 1g, Supplementary Table S4).

Finally, we characterized the activation status of CD4^+^ T cells. As seen in Figure 1g (Supplementary Figure S1d, Supplementary Table S4), we observed an increased level of effector CD4^+^ T cells in the TME of LLC cell tumors. In contrast, the frequencies of memory CD4^+^ T cells were decreased in LLC tumors. The levels of naϊve CD4^+^ T cells were significantly reduced in LLC as compared to KP and MC38 cell tumors, but only moderately reduced (and not significantly) when compared to B16-F10 tumors. No changes were observed for CD44^−^CD62L^−^ CD4^+^ T cells between tumors.

Thus, our data highlight the difference in the lymphoid cell TME between the investigated tumor entities, indicating a low lymphoid cell infiltration into KP tumors, but a strong skew towards CD4^+^ T cell infiltration (Figure 1g). In contrast, B16-F10 tumors show high lymphoid cell infiltration, especially of NKT cells (Figure 1e). Both LLC and MC38 tumors show similar lymphoid cell infiltration; however, the CD4^+^ to CD8^+^ T cell ratio shifts towards CD8^+^ T cells in MC38, while equal frequencies are observed in LLC tumors for these cells.

### 3.3. Subcutaneous Tumors of the Heterotopic Models Exhibit Distinct Compositions of Myeloid Immune Cells within Their TME

**Figure 2:**
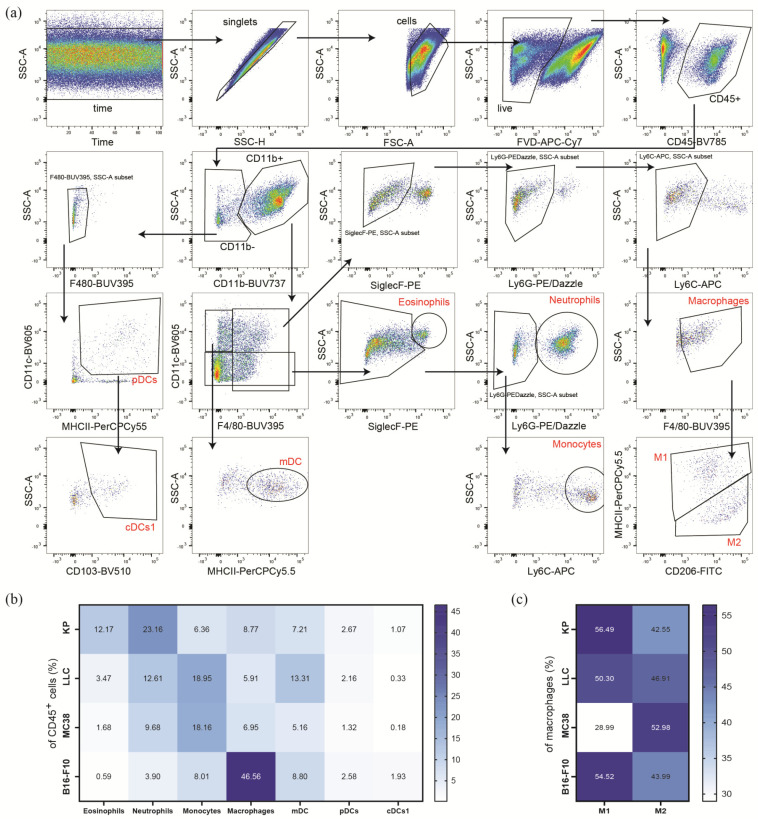
Myeloid immune microenvironment of heterotopic tumor models. (**a**) Representative gating strategy, of KP tumor single-cell suspension, for the analysis of the myeloid immune microenvironment of single-cell suspensions in heterotopic tumors. Tumor infiltrating leukocytes (CD45^+^) were pre-gated for time and singlets. Dead cells were excluded by using a fixable viability dye (FVD). Eosinophils were identified as CD45^+^/CD11b^+^/CD11c^−^/F4/80^+^/Siglec-F^+^; neutrophils as CD45^+^/CD11b^+^/CD11c^−^/Siglec-F^−^/Ly6G^+^; monocytes as CD45^+^/CD11b^+^/CD11c^−^/Siglec-F^−^/Ly6G^−^/Ly6C^+^; and macrophages as CD45^+^/CD11b^+^/CD11c^+/−^/Siglec-F^−^/Ly6G^−^/Ly6C^−^/F4/80^+^. Macrophage subsets were further characterized by MHC-II^+^/CD206^int^ (M1) and MHC-II^int^/CD206^+^ (M2). Dendritic cell (DC) subsets were characterized as follows: mDC (monocyte-derived DC) as CD45^+^/CD11b^+^/CD11c^+^/F4/80^−^/MHC-II^+^, pDC (plasmacytoid DC) as CD45^+^/CD11b^−^/CD11c^+^/MHC-II^+^, and cDCs1 (conventional DC Type 1) as CD45^+^/CD11b^−^/CD11c^+^/MHC-II^+^/CD103^+^. (**b**,**c**) The flow cytometric analysis of single-cell suspensions of s.c. tumors of lung cancer cell lines (KP, *n* = 30; LLC, *n* = 16), colon cancer (MC38, *n* = 28), and melanoma (B16-F10, *n* = 9). Data were pooled from 1 to 4 independent experiments. Heatmaps show percentage of CD45^+^ (**b**) and total macrophages (**c**) ranging from the highest (dark blue) to the lowest numbers (white), respectively.

Next, we used a selection of surface markers to identify myeloid cell populations in the TME of KP, LLC, MC38, and B16-F10 tumors, as depicted in the gating strategy in Figure 2a. As shown in Figure 2b (Supplementary Figure S2a and Supplementary Table S5), we observed an increased frequency of eosinophils in KP cell tumors and of macrophages in B16-F10 tumors when compared to the other tested cell line tumors. Additionally, the KP TME showed double the infiltration of neutrophils compared to those of LLC and MC38 TMEs. The B16-F10 TME was characterized by an even further decrease in infiltrating neutrophils (Figure 2b, Supplementary Figure S2a). With regard to monocytes, we observed a three times increase in frequencies in the TMEs of LLC and MC38 tumors, as compared to KP and B16-F10 tumors (Figure 2b).

Next, we were interested in the abundance of dendritic cell (DC) subsets in the TMEs of the cancer cell lines of interest (Figure 2b, Supplementary Figure S2a, and Supplementary Table S5). We recorded higher frequencies of monocyte-derived DCs (mDCs) in the TME of LLC than in those of KP and B16-F10 tumors. In MC38 tumors, mDCs infiltration was even further reduced (Figure 2b). Plasmacytoid DCs (pDCs) infiltration was lower in the MC38 tumors as compared to the other cell line tumors. With regard to conventional DC Type 1 cells (cDCs1), they were significantly reduced in LLC and MC38 TMEs, when compared to the TMEs of KP and B16-F10 tumors, although cDCs1 frequencies in B16-F10 TMEs were even more increased when compared to the TME of KP tumors. However, it has to be pointed out that pDCs and cDCs1 cells were the least abundant cells in the TMEs of all investigated cell type tumors, as their frequencies were below 3% of CD45^+^ cells.

As a next step, we used antibodies against MHC-II and CD206 to distinguish inflammatory M1 macrophages from anti-inflammatory M2 macrophages (Figure 2c, Supplementary Figure S2b, and Supplementary Table S6). We observed approximately double the frequency of M1 macrophages in KP, LLC, and B16-F10 TMEs compared to the TME of MC38 tumors. A trend towards the increased levels of M2 macrophages was observed in the TME of MC38 tumors; however, there were no significant changes among the investigated TMEs (Figure 2c and Supplementary Table S6).

Collectively, similar to the lymphoid subpopulations, myeloid cell infiltrates differ in their profiles between the tumor entities. LLC and MC38 tumors show similar infiltration of eosinophils, neutrophils, and macrophages. However, LLC tumors tend towards increased DC populations. The TME of KP tumors is highly infiltrated by eosinophils and neutrophils, whereas the TME of B16-F10 tumors shows a strong macrophage infiltration.

## 4. Discussion

In the present study, we provide a comprehensive flow cytometric analysis of the immune TME in the s.c. heterotopic tumor models of lung and colon cancer, as well as melanoma. We used two distinct flow cytometry panels to stain cells of the lymphoid and myeloid populations. We report that cell line-induced tumors widely differ in the immune cell composition of the TME.

Deaths due to cancer are second only to deaths due to cardiovascular diseases [25]. Although immunotherapies have improved the overall survival of cancer patients, these therapies still face limitations, warranting a deeper knowledge of anti-tumor immunity [19]. In this context, tumors in immunocompetent mouse models and the knowledge of their immune TME are not only important to understand the basic mechanisms within the TME but also to investigate the effects of new therapies in cancer.

In our study, we first set out to investigate the immune TME of tumors of two distinct lung cancer cell lines. The used KP cell line was isolated from primary tumors in Kras^LSL−G12D^/p53^fl/fl^ mice after tumor growth was initiated by a Cre-recombinase-expressing adenovirus [23]. We observed that neutrophils were the most prominent immune cells infiltrating the TME of KP cell tumors, which aligns with an article on the TME of human lung cancer types, describing neutrophils as the most abundant cell type in NSCLC [26]. In contrast to our findings, neutrophils were second in abundance after macrophages in a study of orthotopically adenovirus-induced lung cancer [23], possibly because in that model, the virus used for tumor initiation also induced strong inflammation. Nevertheless, Kargl et al. identified an inverse correlation of neutrophils and CD8^+^ T cells in human NSCLC [27], which aligns with our finding of low CD8^+^ T cell infiltration in the TME of KP tumors. A recent preprint describing the mechanisms behind this inverse correlation suggests a role of the neutrophil-derived enzyme myeloperoxidase in suppressing T cell responses [28]. Collectively, our data indicate that the use of KP cells for studying NSCLC is highly translational.

Another lung cancer cell line used in our study was the LLC cells. Monocytes, mDC, neutrophils, and T cells were the most prominent immune cells infiltrating the TME of LLC tumors. In a study from 2013, Lechner and colleagues characterized LLC s.c. tumors as poorly immunogenic, showing the reduced infiltration of anti-tumorigenic effector cells (e.g., CD8^+^ T cells) compared to other investigated cell lines [29]. They identified up to 20% monocyte-derived suppressor cells (MDSC), defined as CD11b^+^Gr-1^+^ (Ly6C^+^/Ly6G^+^) cells, in the TME of LLC tumors [29]. This is in line with our study, in which we detected monocytes (CD11b^+^Ly6C^+^) and neutrophils (CD11b^+^Ly6G^+^) as the major infiltrating immune cell types. Another study revealed that the depletion of these cells with an anti-Gr1 antibody attenuated tumor growth, which was accompanied by a reduction in T regs [30]. Our LLC tumors showed approximately twice the frequency of T cells and mDCs compared to the TME of KP tumors. A mutanome analysis discovered immunogenic neoantigens in LLC cells [31], which could be an explanation for the increased infiltration of T cells and mDCs. An important factor that could explain differences observed in the KP and the LLC TME is the difference in mutational burden. Although both tumor types exhibit KRAS mutations [23,32], they additionally either harbor p53 knockout [23] or mutations in the PI3K-AKT pathway [31]. Indeed, different driver-mutations were described to induce unique immune cell profiles in NSCLC [23]. Finally, we observed similar levels of CD8^+^ and CD4^+^ T cell infiltration and only slightly lower levels of T regs and CD11b^+^Gr-1^+^ (Ly6C^+^/Ly6G^+^) cells in tumors of our heterotopic LLC model, when compared to an orthotopic LLC mouse model [33,34], highlighting the validity and translational value of our models.

We also investigated the TME of MC38-induced s.c. tumors. Here, the most prominent immune cell infiltration was by monocytes, followed by neutrophils, although a strong T cell infiltration was also detected. Jin and colleagues described an immune cell profile of the TME of MC38-derived tumors that was different to ours [35]. The discrepancies are unclear but could be due to the difference in housing conditions. Nevertheless, Zhong et al. characterized MC38 tumors as moderately immunogenic [36]. They compared the MC38 heterotopic tumor model to human colorectal cancer by using gene expression analysis and detected similar cytolytic activity [36]. MC38 tumors also showed a good response to anti-CTLA4 antibodies, but only a moderate response to anti-PD-1 checkpoint inhibitor treatment [36]. In contrast to the MC38 model, the colorectal cancer cell line CT26 shows high immunogenicity, high cytolytic activity, and a strong response to checkpoint inhibitor treatment [36]. Additionally, researchers have used genetic or inflammation-induced orthotopic tumor models for investigating colorectal cancer [24,37,38]. When comparing the frequencies of immune cells in the TME of our heterotopic MC38 tumors with these models [24,37], we noticed similar levels of eosinophils and macrophages. Only the inflammation-mediated AOM/DSS model showed an enhanced infiltration of neutrophils and CD3^+^ cells [24,38]. Here, it should be highlighted that the microbiota plays an important role in the inflammation-driven tumor models of colorectal cancer, influencing immune cell infiltration and tumor growth [39]. Notably, neutrophil, monocyte, and macrophage levels in our MC38 model are quite comparable to those of genetic colorectal cancer models (APC^Min/+^) [37,40]. Furthermore, recent studies observed diverse TME clusters (including high or low immune infiltration) that correlated with tumor mutational burden and patient survival in different RNAseq data sets of cohorts of CRC [41,42]. Thus, our data support the use of the s.c. MC38-induced tumor model, e.g., for the investigation of new anti-tumorigenic agents that could shift a “cold” colorectal tumor to a “hot” colorectal tumor.

Recently, a report was released indicating that deaths from melanoma will increase by about 68% from 2020 to 2040 [43]; thus, an s.c. model using B16-F10 melanoma cells was included in our study. Although the viability of B16-F10 tumor single-cell suspensions was only about 5%, we could show that macrophages were the most prominent immune cell types infiltrating the B16-F10 cell TME, and that they were skewed towards M1 macrophages. They were followed by T cells, even though Zhong et al. characterized B16-F10 tumors as low immunogenic, with the lowest cytolytic activity and no response to anti-CTLA4 or anti-PD1 checkpoint inhibitor therapies [36]. In contrast to a study by Lucarini and colleagues, we observed a 10-fold higher infiltration of CD45^+^ cells, a 4-fold higher frequency in macrophages, and reduced T cell frequencies in our melanomas, but similar levels of infiltrating eosinophils [44]. As a downside of the s.c. melanoma model, a low B16-F10 immunogenicity may not reflect the human picture of melanoma in which the cytolytic activity is moderate [36]. Huang et al. correlated the infiltration of various immune cell types in human melanoma with the survival of patients [45]. They found an improved survival with reduced M2 infiltration and an increased M1 macrophage presence, which we also noted in our s.c. tumors. They additionally showed the inverse correlation of M2 macrophages with CD8^+^ T cells [45]. Finally, we observed a prominent NKT cell infiltration into the B16-F10 TME. The importance of NKT cells for allogenic cancer cell therapy has only recently emerged [46]. Thus, our data of B16-F10-derived melanoma could, to a certain extent, support the use of heterotopic melanoma models for the research of the immune TME and translational research with cancer immunotherapies.

The use of heterotopic instead of orthotopic tumor models is an obvious limitation of our study. However, even though these models do not reflect the tumor in its natural environment, they have become an integral part of cancer research [47]. The benefits of these models include the ease of engraftment and their time/cost effectiveness, unlike the complex induction of tumor growth in other models (chemically or by surgery/injection) [47]. They are easy to perform, and if very large tumors are not grown, the experimental burden on animals is relatively low. However, an important caveat is that driver-mutations in mouse cancer cell lines can differ from those in human cancer cells. As such, the common APC mutation in colorectal cancer is not detected in MC38, nor in CT26 cells [36]. On the other hand, TP53 mutations are found in experimental cell lines of lung cancer and melanoma as well as in their human counterparts [36]. With regard to lung cancer, KRAS driver-mutations are found in both KP and LLC tumors [23,32,36]. These facts have to be considered when choosing the right tumor model, as driver-mutations reportedly affect immune cell infiltration, like in lung cancer [23] and melanoma [48].

Heterotopic tumor models are frequently used to investigate ways of influencing the TME. Recent publications showcase the use of the KP cell line as means to examine the possibility of switching a “cold” tumor into a “hot” tumor [49,50]. In this context, our group showed an improved response to PD-1 therapy in mice bearing KP cell tumors in which the cannabinoid-receptor 2 was absent in the TME [49]. In MC38 tumors, the depletion of CD4^+^ T cells, CD25^+^ (T regs), or macrophages reduced tumor growth and supported PD-1 therapy [35]. Furthermore, T reg depletion or IL-33 treatment enhanced eosinophil infiltration into B16-F10 tumors and reduced tumor growth [44,51]. The agonism of 4-1BB (also named CD137; a costimulatory receptor of the tumor necrosis factor receptor superfamily) boosted PD-1 therapy in B16-F10 [52].

## 5. Conclusions

To conclude, our study provides a detailed overview of the immune TME in heterotopic tumor models of lung and colon cancer, and of melanoma. By using multicolor flow cytometry, we were able to decipher the lymphoid and myeloid immune cell composition of their TMEs. Our data highlight the translational value of these models for research on new immunotherapies. Certainly, they can be a guide for the choice of the appropriate tumor model when investigating the immune TME of lung, colon, and skin cancer.

## Data Availability

Data are contained within the article or supplementary materials.

## Supplementary Materials

The following supporting information can be downloaded at: https://www.mdpi.com/article/10.3390/cancers16020295/s1, Supplementary Figure S1: Lymphoid immune composition depicted as absolute cells/mg tumor tissue; Supplementary Figure S2: Myeloid immune composition depicted as absolute cells/mg tumor tissue; Supplementary Table S1: Statistical differences of % of lymphoid cells of CD45^+^ cells in the tumor microenvironment of indicated cell lines; Supplementary Table S2: Statistical differences of % of CD3^+^ cells in the tumor microenvironment of indicated cell lines; Supplementary Table S3: Statistical differences of % of CD8^+^ cells in the tumor microenvironment of indicated cell lines; Supplementary Table S4: Statistical differences of % of CD4^+^ cells in the tumor microenvironment of indicated cell lines; Supplementary Table S5: Statistical differences of % of myeloid cells of CD45^+^ cells in the indicated tumor microenvironment of cell lines; Supplementary Table S6: Statistical differences of % macrophages cells in the tumor microenvironment of indicated cell lines.
