# Supplementary material for: Comparative Study of the Immune Microenvironment in Heterotopic Tumor Models
Source: Cancers (Basel). 2024 Jan 10;16(2):295. doi: 10.3390/cancers16020295 (PMC10813609; doi:10.3390/cancers16020295)
Supplement: Supplementary file 1 [file cancers-16-00295-s001.zip › cancers-2807882-supplementary.pdf]

Supplementary Material

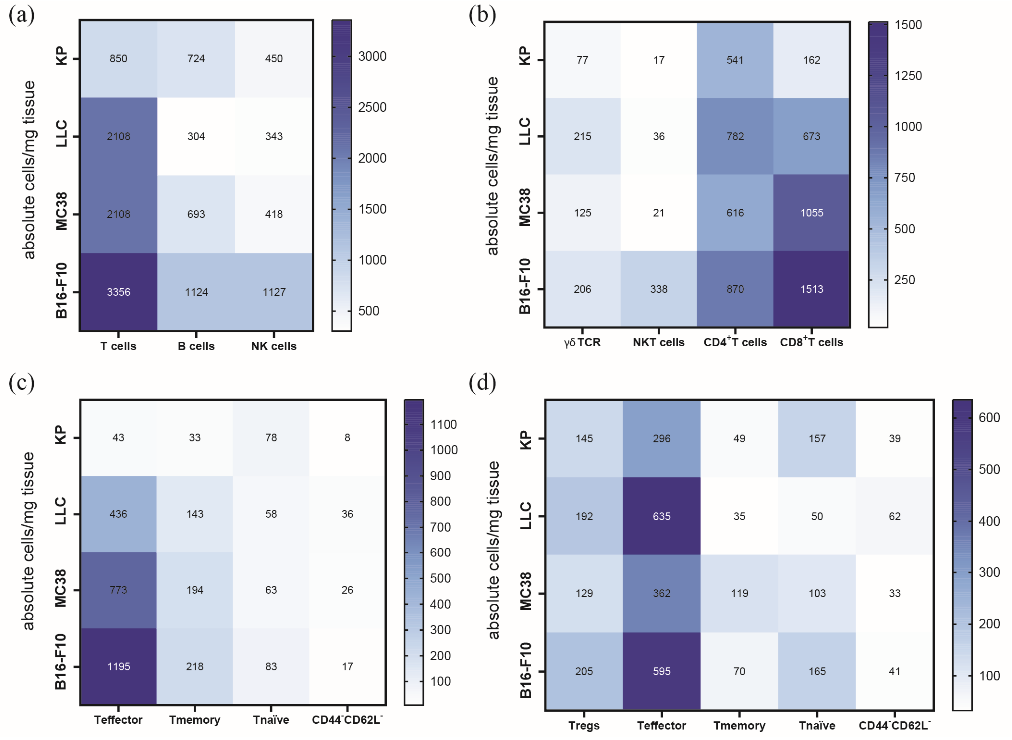

Supplementary Figure S1. Lymphoid immune composition depicted as absolute cells/mg tumor tissue. (**a–d**) Flow cytometric analysis of single-cell suspensions of s.c. tumors of lung cancer cell lines (KP, n=32; LLC, n = 18), colon cancer (MC38, n = 28), and melanoma (B16-F10, n = 17). Data were pooled from 2 to 5 independent experiments. Heatmaps show absolute cells per mg tumor tissue of lymphoid (a), $CD3^+$ (b), $CD8^+$ (c), and $CD4^+$ (d) cells measured in tumors, ranging from the highest (dark blue) to the lowest (white) number, respectively.

Supplementary Table S1. Statistical differences of % of lymphoid cells of $CD45^+$ cells in the tumor microenvironment of indicated cell lines. Significant differences between four experimental groups were determined using one-way ANOVA with Tukey's post hoc test for corrections of multiple comparisons. **p<0.01; ***p<0.001; ****p<0.0001; n.s., non-significant; and NK cells, natural killer cells.

**% of CD45⁺ cells**	**KP vs. LLC**	**KP vs. MC38**	**KP vs. B16-F10**	**LLC vs. MC38**	**LLC vs. B16-F10**	**MC38 vs. B16-F10**
$CD3^+$ cells	****	***	****	n.s.	**	****
B cells	n.s.	n.s.	n.s.	n.s.	n.s.	n.s.
NK cells	n.s.	n.s.	n.s.	n.s.	n.s.	n.s.

Supplementary Table S2. Statistical differences of % of $CD3^+$ cells in the tumor microenvironment of indicated cell lines. Significant differences between four experimental groups were determined using one-way ANOVA with Tukey's post hoc test for corrections of multiple comparisons. *p<0.05; ***p<0.001; ****p<0.0001; n.s., non-significant; and NKT cells, natural killer T cells.

% of $CD3^+$ cells	KP vs. LLC	KP vs. MC38	KP vs. B16-F10	LLC vs. MC38	LLC vs. B16-F10	MC38 vs. B16-F10
$\gamma\delta TCR^+$ T cells	n.s.	***	****	***	****	n.s.
NKT cells	n.s.	n.s.	****	n.s.	****	****
$CD4^+$ T cells	****	****	****	n.s.	n.s.	n.s.
$CD8^+$ T cells	****	****	****	****	*	n.s.

Supplementary Table S3. Statistical differences of % of $CD8^+$ cells in the tumor microenvironment of indicated cell lines. Significant differences between four experimental groups were determined using one-way ANOVA with Tukey's post hoc test for corrections of multiple comparisons. *p<0.05; ****p<0.0001; and n.s., non-significant.

% of $CD8^+$ cells	KP vs. LLC	KP vs. MC38	KP vs. B16-F10	LLC vs. MC38	LLC vs. B16-F10	MC38 vs. B16-F10
T effector	****	****	****	n.s.	n.s.	n.s.
T memory	n.s.	n.s.	n.s.	n.s.	n.s.	n.s.
T naïve	****	****	****	n.s.	n.s.	n.s.
$CD44^-CD62L^-$	n.s.	n.s.	*	n.s.	n.s.	n.s.

Supplementary Table S4. Statistical differences of % of $CD4^+$ cells in the tumor microenvironment of indicated cell lines. Significant differences between four experimental groups were determined using one-way ANOVA with Tukey's post hoc test for corrections of multiple comparisons. *p<0.05; **p<0.01; ****p<0.0001; n.s., non-significant; and T regs, regulatory T cells

% of $CD4^+$ cells	KP vs. LLC	KP vs. MC38	KP vs. B16-F10	LLC vs. MC38	LLC vs. B16-F10	MC38 vs. B16-F10
T regs	n.s.	****	n.s.	**	n.s.	****
T effector	****	n.s.	n.s.	****	**	n.s.
T memory	*	**	n.s.	****	****	n.s.
T naïve	**	n.s.	n.s.	**	n.s.	n.s.
$CD44^-CD62L^-$	n.s.	n.s.	n.s.	n.s.	n.s.	n.s.

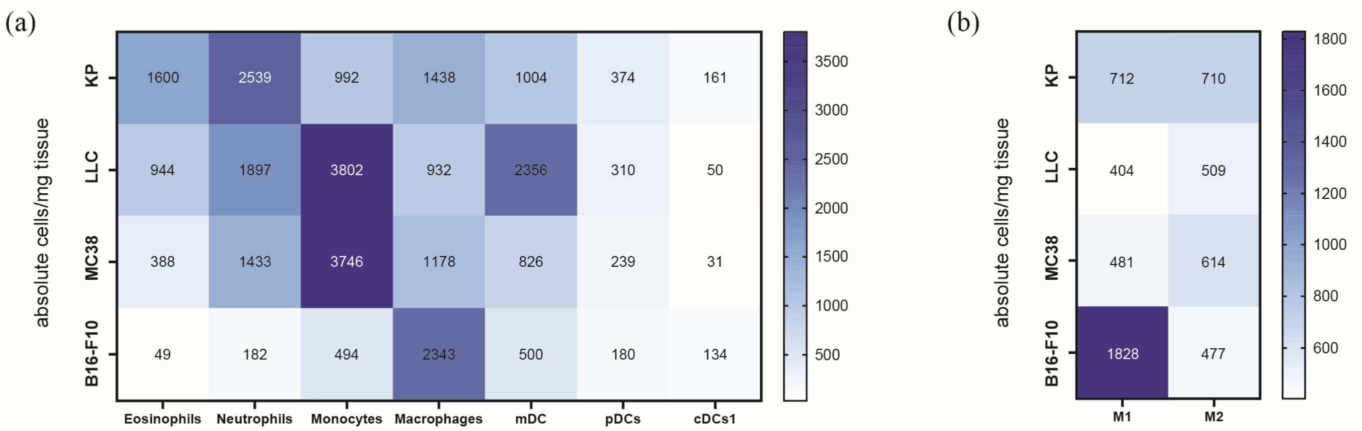

Supplementary Figure S2. Myeloid immune composition depicted as absolute cells/mg tumor tissue. (**a**,**b**) Flow cytometric analysis of single-cell suspensions of s.c. tumors of lung cancer cell lines (KP, n=30; LLC, n=16), colon cancer (MC38, n=28), and melanoma (B16-F10, n=9). Data were pooled from 1 to 4 independent experiments. Heatmaps show absolute cells per mg tumor tissue of myeloid (**a**) and total macrophages (b), ranging from the highest (dark blue) to the lowest (white) numbers, respectively.

Supplementary Table S5. Statistical differences of % of myeloid cells of CD45$^+$ cells in the indicated tumor microenvironment of cell lines. Significant differences between four experimental groups were determined using one-way ANOVA with Tukey's post hoc test for corrections of multiple comparisons. *p<0.05; **p<0.01; ***p<0.001; ****p<0.0001; n.s, non-significant; mDC, monocyte-derived dendritic cell; pDC, plasmacytoid dendritic cells; and cDCs1, conventional dendritic cells type 1

**% of CD45$^+$ cells**	**KP vs. LLC**	**KP vs. MC38**	**KP vs. B16-F10**	**LLC vs. MC38**	**LLC vs. B16-F10**	**MC38 vs. B16-F10**
Eosinophils	****	****	****	n.s.	n.s.	n.s.
Neutrophils	****	****	****	n.s.	*	n.s.
Monocytes	****	****	n.s.	n.s.	****	****
Macrophages	n.s.	n.s.	****	n.s.	****	****
mDCs	****	*	n.s.	*	***	**
pDCs	n.s.	****	n.s.	*	n.s.	**
cDCs1	***	****	**	n.s.	****	****

Supplementary Table S6. Statistical differences of % macrophages cells in the tumor microenvironment of indicated cell lines. Significant differences between four experimental groups were determined using one-way ANOVA with Tukey's post hoc test for corrections of multiple comparisons. **p<0.01; ***p<0.001; ****p<0.0001; and n.s., non-significant.

**% macrophages**	**KP vs. LLC**	**KP vs. MC38**	**KP vs. B16-F10**	**LLC vs. MC38**	**LLC vs. B16-F10**	**MC38 vs. B16-F10**
M1 macrophages	****	n.s.	n.s.	***	n.s.	**
M2 macrophages	n.s.	n.s.	n.s.	n.s.	n.s.	n.s.
